# Regional variation in low-value musculoskeletal surgery: a nationwide study from the Finnish Care Register

**DOI:** 10.2340/17453674.2024.42413

**Published:** 2024-11-11

**Authors:** Ville PONKILAINEN, Anniina LAUREMA, Ville M MATTILA, Teemu KARJALAINEN

**Affiliations:** 1 Department of Orthopaedics and Traumatology, Tampere University Hospital; 2 Department of Surgery, Mikkeli Central Hospital, Mikkeli; 3 COXA Hospital for Joint Replacement, Tampere; 4 Faculty of Medicine and Life Sciences, University of Tampere, Tampere; 5 Department of Surgery, Central Finland Central Hospital, Jyväskylä, Finland

We would like to inform of an error in the reported incidences in our published article, *Regional variation in low-value musculoskeletal surgery: a nationwide study from the Finnish Care Register*. Specifically, the incidences for rotator cuff repair, partial meniscectomy, ankle arthroscopy, distal radius fracture fixation, and wrist arthroscopy surgeries were calculated using a too small population size.

Accordingly, corrected calculations resulted in higher incidence values for these surgeries, impacting Table 3 and Figure 3, which now show accurate incidence rates. We have also updated Figure 6 and the corresponding sections of the results accordingly.

These corrections did not affect any other figures or tables, not the overall conclusions, and the text in the discussion section remains unchanged. The correct data further emphasizes the findings in our original article.

We apologize for any confusion caused by these errors and appreciate the opportunity to correct the publication.

*Corrected version*

## Results

The total number of low-value surgeries declined from 31,824 in 2006–2007 to 6,627 (–79%) in 2020–2021 (Figure 1). Within the 20 hospital districts, the median incidence was 15 per 10^5^ person-years (range 7–40, IQR 12–16).

In public hospitals, the total incidence of low-value surgeries ranged between 3 and 35 per 10^5^ person-years, while in private hospitals, the incidence ranged between 2 and 13 (Figure 2, Table 2). In Central Ostrobothnia, Kainuu, Länsi Pohja, South Karelia or South Savo Hospital Districts no low-value surgeries were performed in private hospitals likely due to no private service providers in the districts.

**Figure 3:**
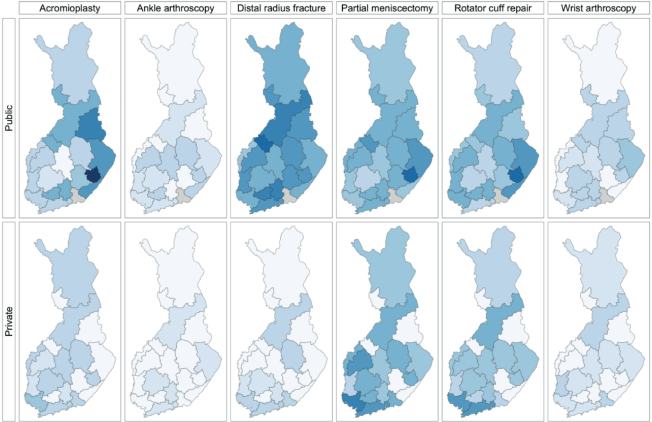
Total incidence (per 10^5^ person-years) of low-value surgeries by hospital districts in Finland in 2020–2021, divided by surgery, separately by public and private sectors. For name of hospital districts, see Figure 2 and Table 2.

The most commonly performed low-value surgeries were partial meniscectomies and rotator cuff repairs in East Savo public hospitals by 88 (CI 56–130) and 86 (CI 43–150) per 10^5^ person-years, respectively (Figure 3, Table 3, see Appendix).

*Old version*

## Results

The total number of low-value surgeries declined from 31,824 in 2006–2007 to 6,627 (–79%) in 2020–2021 (Figure 1). Within the 20 hospital districts, the median incidence was 15 per 10^5^ person-years (range 7–40, IQR 12–16).

In public hospitals, the total incidence of low-value surgeries ranged between 3 and 35 per 10^5^ person-years, while in private hospitals, the incidence ranged between 2 and 13 (Figure 2, Table 2). In Central Ostrobothnia, Kainuu, Länsi Pohja, South Karelia, or South Savo Hospital Districts no low-value surgeries were performed in private hospitals likely due to no private service providers in the districts.

The most commonly performed low-value surgeries were acromioplasties and partial meniscectomies in East Savo public hospitals at 67 (CI 43–101) per 10^5^ person-years for both, and partial meniscectomies in private Southwest Hospital District 48 (CI 41–55, change +58%) per 10^5^ person-years (Figure 3, Table 3, see Appendix).

Corrected version

**Table 1:** NOMESCO procedure codes, diagnosis codes, age limitations, and certainty of evidence for all evaluated surgeries

Surgery/NOMESCO code	Diagnosis codes	Age, years	Evidence	Certainty of evidence
Acromioplasty NBG10 Acromioplasty NBG15 Acromioplasty, arthroscopic	M* Diseases of the musculoskeletal system and connective tissue.	> 18	Little or no benefit	High, 8 trials [7]
Partial meniscectomy NGD05 Partial excision of meniscus of knee, arthroscopic	S* Injury, poisoning and certain other consequences of external causes.	> 18 > 40	Little or no benefit No supporting evidence ^a^	High, 16 trials [24] NA ^a^ [25]
Wrist arthroscopy NDF25 Operation for osteochondritis of joint of wrist, arthroscopic Ankle arthroscopy NHA30 Exploration of joint of ankle or foot, arthroscopic NHF* Operations on synovia and joint surfaces of ankle and foot Rotator cuff repair NBL00 Suture or reinsertion of rotator cuff NBL05 Arthroscopic suture or reinsertion of rotator cuff	T9* Sequelae of injuries, of poisoning and of other consequences of external causes	> 40 > 65	No supporting evidence ^a^ Clinically unimportant benefit	NA ^a^ [26] NA ^a^ [6]
Distal radius fracture fixation NDJ62 Internal fixation of fracture of wrist or hand using plate and screws NCJ62 Internal fixation of fracture of forearm using plate	S52.5 Fracture of lower end of radius S52.4 Fracture of shafts of both ulna and radius	> 65	Clinically unimportant compared with cast in people > 60 ^b^	High, 12 trials [27]

a No trials comparing surgery and nonoperative treatment or placebo.

b Evidence is limited only to distal radius fractures with dorsal displacement (Colles).

NA = not applicable.

*Corrected version*

## Factors describing the regional variation

The incidence in years 2006–2007 had a positive correlation with the incidence in 2020–2021 (r = 0.69, CI 0.37–0.87) (Figure 4). A Poisson regression model, adjusted for the mean population size, showed a β = 0.009 (CI 0.006–0.012), indicating an increase in the incidence of low-value surgery with each unit increase in the incidence of reference years.

There was a negative correlation between the incidence of private and public hospitals (r = –0.43, CI –0.73 to 0.001] (Figure 5). A Poisson regression model, adjusted for the incidence in the reference years (2006–2007), showed a β = –0.03 (CI –0.04 to 0.012), indicating a reduction in the incidence of low-value surgery with each unit increase in the incidence in private hospitals.

A negative correlation existed between the incidence in the public hospitals and mean population size (r = –0.34, CI –0.68 to 0.12) (Figure 6). A Poisson regression model, adjusted for the incidence in the reference years (2006–2007), showed a β = –0.5 (CI –2.03 to 1.00), indicating a reduction in the incidence of low-value surgery with each unit increase in mean population size.

*Old version*

## Factors describing the regional variation

The incidence in years 2006–2007 had a positive correlation with the incidence in 2020–2021 (r = 0.69, CI 0.37–0.87; Figure 4). A Poisson regression model, adjusted for the mean population size, showed β = 0.015 (CI 0.01–0.02), indicating an increase in the incidence of low-value surgery with each unit increase in the incidence of reference years.

There was a negative correlation between the incidence of private and public hospitals (r = –0.43, CI –0.73 to 0.001; Figure 5). A Poisson regression model, adjusted for the incidence in the reference years (2006–2007), showed β = –0.04 (CI –0.08 to 0.004), indicating a reduction in the incidence of low-value surgery with each unit increase in the incidence in private hospitals.

A negative correlation existed between the incidence in the public hospitals and mean population size (r = –0.42, CI –0.72 to 0.02; Figure 6). A Poisson regression model, adjusted for the incidence in the reference years (2006–2007), showed β = –0.04 (CI –0.09 to 0.01), indicating a reduction in the incidence of low-value surgery with each unit increase in mean population size.

*Corrected version*

**Figure:**
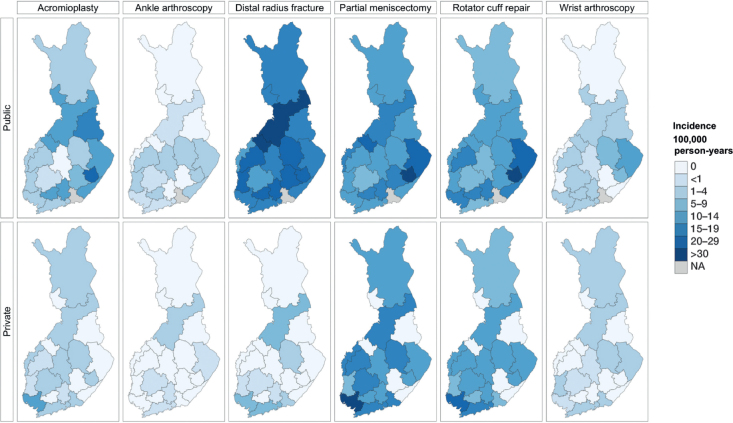


*Old version*

*Corrected version*

**Figure 6:**
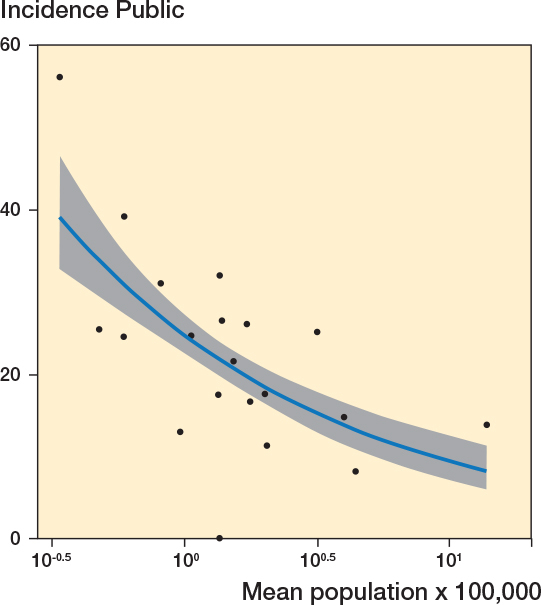
Correlation between the incidence of low-value surgeries in public hospitals and mean population size per region (r = –0.34, CI –0.68 to 0.12). Also see legend to Figure 4.

*Old version*

**Figure 6:**
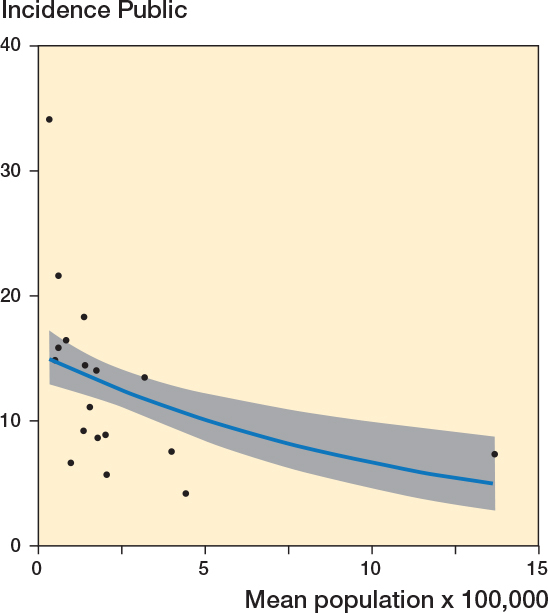
Correlation between the incidence of low-value surgeries in public hospitals and mean population size per region (r = –0.42, CI –0.72 to –0.02). Also see legend to Figure 4.

*Corrected version*

**Table 3:** Incidence of low-value care based on hospital district, divided into years 2006–2007 and 2020–2021 by hospital type (private vs public)

Procedure Hospital district ^a^	Private hospitals		Change (%)	Public hospitals		Change (%)
	2006–2007	2020–2021		2006–2007	2020–2021	
Acromioplasty						
1	14 (9.2–20)	2.5 (0.8–5.8)	–82	72 (61–85)	–	–100
2	–	–	–	120 (92–150)	19 (9.9–34)	–84
3	–	3 (0.08–17)	–	350 (300–420)	68 (43–100)	–81
4	26 (23–29)	3 (2.1–4)	–89	87 (81–92)	4.6 (3.5–5.9)	–95
5	–	–	–	95 (73–120)	33 (20–52)	–65
6	9.4 (4.9–16)	–	–100	110 (95–130)	13 (8–21)	–88
7	0.71 (0.02–4)	1.9 (0.31–6)	160	110 (89–120)	–	–100
8	–	1.6 (0.11–6.7)	–	140 (110–160)	1 (0.03–5.8)	–99
9	–	–	–	250 (210–290)	20 (9.4–37)	–92
10	37 (27–48)	–	–100	200 (170–220)	29 (20–39)	–86
11	50 (42–59)	2.9 (1.3–5.5)	–94	88 (77–100)	11 (8.1–16)	–87
12	18 (13–25)	4 (1.7–7.9)	–78	250 (230–270)	1.5 (0.31–4.4)	–99
13	32 (27–38)	2.9 (1.5–4.9)	–91	98 (89–110)	0.46 (0.06–1.7)	–100
14	1.5 (0.25–4.8)	0.29 (0.00–2.7)	–80	90 (76–110)	11 (6.9–18)	–87
15	12 (7.4–18)	0.86 (0.06–3.7)	–93	110 (94–120)	3.1 (1.1–7.1)	–97
16	9.8 (4.8–18)	–	–100	180 (160–210)	25 (17–37)	–86
17	11 (6.7–18)	0.99 (0.07–4.2)	–91	110 (91–120)	4 (1.5–8.6)	–96
18	–	–	–	130 (110–160)	6.8 (2.4–15)	–95
19	32 (26–38)	10 (7.2–14)	–68	160 (150–170)	1.4 (0.48–3.1)	–99
20	7.6 (3.5–14)	1.9 (0.31–6)	–75	60 (47–75)	3.8 (1.2–8.8)	–94
Ankle arthroscopy						
1	–	–	–	2.5 (0.51–7.2)	3.8 (1.2–8.9)	55
2	–	–		5.2 (0.63–19)	–	
3	–	–	–	7.1 (0.86–26)	3.8 (0.1–21)	–46
4	2.2 (1.2–3.6)	0.36 (0.08–1.1)	–84	2.1 (1.2–3.5)	0.66 (0.23–1.5)	–69
5	–	–		2.2 (0.06–12)	–	
6	–	–	–	6.6 (2.4–14)	2.5 (0.42–8.1)	–62
7	–	–		2 (0.24–7.3)	–	
8	–	–		3.8 (0.64–12)	–	
9	–	–	–	–	–	53
10	–	0.53 (0.00–5)	–	1 (0.03–5.8)	1.6 (0.12–6.9)	53
11	–	1.3 (0.21–4)	–	3.2 (1.1–7.1)	0.25 (0.00–2.3)	–92
12	–	–	–	4.1 (1.4–9.2)	2.6 (0.62–7)	–37
13	0.2 (0.00–1.9)	0.36 (0.01–2)	79	2.2 (0.77–5)	0.72 (0.087–2.6)	–68
14	–	0.82 (0.02–4.6)	–	2.6 (0.54–7.6)	–	–100
15	–	–	–	1.6 (0.19–5.8)	2 (0.33–6.4)	25
16	–	–	–	3.3 (0.55–11)	4 (0.82–12)	20
17	–	–	–	3.8 (1.0–9.7)	2.8 (0.58–8.2)	–26
18	–	–		3.2 (0.38–11)	–	
19	0.63 (0.05–2.7)	–	–100	1.5 (0.36–4)	0.77 (0.09–2.8)	–48
20	–	–		2.4 (0.3–8.8)	–	
Distal radius fracture						
1	–	–	–	18 (7.3–37)	39 (24–59)	110
2	–	–	–	8 (0.2–44)	82 (45–140)	930
3	–	–		–	59 (24–120)	
4	0.29 (0.00–2.7)	9.2 (6–13)	3,100	29 (21–38)	46 (38–54)	59
5	–	–	–	10 (0.72–43)	40 (17–80)	300
6	–	–		–	66 (43–95)	
7	–	4.5 (0.55–16)	–	7.7 (1.3–25)	–	–100
8	–	–	–	25 (8–57)	38 (18–68)	53
9	–	–	–	26 (5.5–77)	45 (18–92)	69
10	–	–	–	21 (8.2–45)	39 (23–63)	84
11	–	8.3 (3.2–18)	–	51 (34–75)	75 (57–97)	46
12	–	3.3 (0.41–12)	–	8 (1.9–22)	52 (35–74)	550
13	0.63 (0.00–5.9)	0.87 (0.02–4.8)	37	24 (14–38)	27 (18–38)	12
14	–	0.92 (0.00–8.6)	–	9.6 (2.3–26)	62 (42–86)	540
15	–	0.88 (0.00–8.2)	–	4.7 (0.57–17)	53 (36–75)	1000
16	–	–	–	12 (2.4–35)	36 (18–62)	200
17	–	–	–	7 (1.2–22)	55 (36–80)	680
18	–	–	–	6.9 (0.5–30)	58 (33–93)	730
19	–	5.9 (2.3–13)	–	32 (21–48)	34 (24–47)	6.1
20	–	–	–	27 (11–54)	20 (8.3–40)	–26
Partial meniscectomy						
1	44 (33–58)	13 (7.3–20)	–72	420 (390–460)	15 (9–23)	–96
2	–	–	–	410 (340–470)	57 (36–85)	–86
3	–	21 (7.3–47)	–	370 (310–450)	88 (56–130)	–77
4	140 (130–150)	49 (44–54)	–66	220 (210–230)	20 (17–23)	–91
5	–	–	–	240 (200–290)	24 (12–44)	–90
6	36 (25–51)	28 (19–41)	–21	380 (340–420)	30 (21–43)	–92
7	13 (7–22)	37 (26–52)	180	180 (150–210)	–	–100
8	–	15 (7.3–28)	–	290 (250–330)	11 (4.3–22)	–96
9	–	–	–	180 (140–230)	29 (14–53)	–84
10	53 (39–69)	13 (6.6–22)	–76	190 (170–220)	50 (37–67)	–74
11	140 (130–160)	31 (24–39)	–79	290 (260–310)	36 (28–45)	–87
12	46 (36–59)	40 (30–52)	–13	270 (250–300)	20 (14–30)	–93
13	220 (200–240)	32 (26–40)	–85	300 (280–330)	8.6 (5.5–13)	–97
14	63 (50–80)	18 (11–27)	–72	240 (220–270)	38 (28–50)	–85
15	82 (67–100)	9.6 (4.9–17)	–88	220 (190–240)	18 (12–28)	–92
16	91 (70–110)	–	–100	200 (170–240)	15 (7.8–27)	–92
17	36 (26–50)	41 (29–55)	12	300 (270–330)	28 (19–41)	–90
18	0.79 (0.00–7.4)	–	–100	230 (190–270)	37 (23–56)	–84
19	180 (170–200)	73 (63–85)	–60	380 (350–400)	11 (7.6–16)	–97
20	53 (38–71)	40 (27–55)	–25	130 (100–150)	36 (24–50)	–72
Rotator cuff repair						
1	10 (2.8–27)	12 (4.5–25)	13	27 (13–49)	10 (3.5–22)	–64
2	–	–	–	84 (41–150)	17 (3.5–49)	–80
3	–	12 (0.84–50)	–	120 (62–210)	86 (43–150)	–28
4	42 (33–52)	40 (33–48)	–3.1	45 (36–56)	9.4 (6.2–14)	–79
5	–	–	–	23 (5.6–64)	10 (1.2–36)	–57
6	–	7.2 (1.5–21)	–	24 (9.6–49)	32 (17–54)	34
7	–	6.8 (1.4–20)	–	63 (39–97)	–	–100
8	–	7.2 (0.87–26)	–	65 (35–110)	9 (1.5–29)	–86
9	–	–	–	13 (0.96–57)	19 (4–56)	43
10	25 (10–49)	11 (3.2–26)	–57	72 (45–110)	53 (34–80)	–26
11	24 (12–41)	24 (14–37)	–0.67	40 (24–61)	24 (14–37)	–40
12	22 (10–41)	19 (9.8–34)	–11	130 (100–170)	21 (11–36)	–84
13	16 (8.3–27)	13 (7.3–21)	–18	27 (17–41)	6.5 (2.7–13)	–76
14	11 (3–28)	15 (6.3–29)	33	47 (27–75)	31 (18–50)	–33
15	4.7 (0.57–17)	7.1 (1.9–18)	49	40 (23–64)	24 (13–40)	–41
16	16 (4.3–40)	–	–100	77 (47–120)	43 (24–72)	–44
17	13 (3.8–31)	17 (7.3–33)	34	34 (17–59)	38 (22–60)	13
18	–	–	–	99 (62–150)	14 (3.8–36)	–86
19	44 (30–62)	50 (38–65)	14	58 (42–78)	33 (23–45)	–43
20	5.4 (0.39–23)	11 (2.9–27)	98	13 (3–34)	11 (2.9–27)	–15
Wrist arthroscopy						
1	0.41 (0.00–3.8)	0.38 (0.00–3.6)	–6.7	6.5 (2.8–13)	0.38 (0.00–3.6)	
2	–	–		–	2.5 (0.06–14)	
3	–	–	–	16 (4.8–39)	5.7 (0.41–25)	
4	1.2 (0.55–2.4)	2 (1.2–3.2)	60	2.4 (1.4–3.9)	2.3 (1.4–3.6)	
5	–	–		–	4.6 (0.56–17)	
6	–	–		–	4 (1.1–10)	
7	–	2.5 (0.42–8.1)	–	4.5 (1.4–11)	–	
8	–	1.5 (0.04–8.5)	–	1.5 (0.04–8.6)	–	
9	–	–	–	7 (1.2–22)	2.9 (0.07–16)	
10	1.6 (0.11–6.7)	1.6 (0.12–6.9)	1.6	7.4 (3–15)	20 (12–31)	
11	4.6 (2–9.1)	3.5 (1.4–7.2)	–24	13 (7.9–19)	4 (1.7–7.9)	
12	–	1.5 (0.18–5.3)	–	2.2 (0.46–6.5)	7.7 (3.8–14)	
13	1.2 (0.25–3.5)	0.36 (0.01–2)	–70	4.2 (2.1–7.6)	2.3 (0.89–4.9)	
14	0.88 (0.0–4.9)	–	–100	4.4 (1.4–10)	2 (0.34–6.6)	
15	2.4 (0.49–7)	3.2 (0.87–8.2)	34	0.4 (0.00–3.7)	–	
16						
17	–	–	–	1.4 (0.1–6.1)	1.4 (0.1–6)	
18						
19	0.42 (0.01–2.4)	0.19 (0.00–1.8)	–55	–	1.1 (0.24–3.4)	
20	–	–		–	–	

a For name of hospital districts, see Table 2. NA = not available.

